# Employee Grievance Redressal and Corporate Ethics: Lessons from the Boeing 737-MAX Crashes

**DOI:** 10.1007/s11948-024-00475-3

**Published:** 2024-04-11

**Authors:** Shreesh Chary

**Affiliations:** Symbiosis School of Economics, Symbiosis International (Deemed University) (SIU), S.B. Road, Pune, India

**Keywords:** Boeing, Federal aviation administration, Employee grievance redressal, Regulatory capture, Revolving door

## Abstract

Two Boeing 737-MAX passenger planes crashed in October 2018 and March 2019, suspending all 737-MAX aircraft. The crashes put Boeing’s corporate practices and culture under the spotlight. The main objective of this paper is to use the case of Boeing to highlight the importance of efficient employee grievance redressal mechanisms and an independent external regulator. The methodology adopted is a qualitative analysis of statements of various whistleblowers and Boeing and the Federal Aviation Administration (FAA) stakeholders. It suggests that employee feedback flowing up the chain of command should be more flexible and dealt with more seriousness. It recommends that companies adopt a cooling-off period or a lifetime restriction for employees who have gone through the revolving door between regulators and the industry. The Boeing 737-MAX case, which emphasizes the ethical obligations of the job, can offer value to engineers, engineering educators, managers, ombudsmen, and human resource professionals.

## Introduction

Effective employee relations management and grievance resolution are vital elements of contemporary enterprises, crucial for organizations’ performance and their personnel’s welfare. Employment relations have changed due to the deterioration of collective labor relations and the individualization of the working relationship (Walker & Hamilton, 2011). In this context, any enterprise must be able to address grievances raised by employees in the workplace. McCabe and Lewin (1992) state that there are two elements to employee “voice” or employee grievances. The first is when workers voice their objections to management in a work-related setting, mainly when those issues are significant enough to justify filing official grievances. The second, more modern of the two, entails employee involvement in the decision-making processes of the business organization. Ogbonnaya et al. (2022) state that employer-employee or manager-employee relationships are typically based on mutual respect, trust, and support; however, using cost-cutting measures breeds insecurity, which stifles the overall spirit of cooperation in the workplace.

The problem of ignoring employee grievances is further exacerbated by regulatory capture (Stigler, 1971), which afflicts many industries and their respective regulators. Regulatory capture is a situation in which certain entities influence the behavior of regulatory agencies in diverse areas, such as legislation, finances, and taxation (Dal Bo, 2006). It is a method by which a body furthers the particular interests of the sectors and other entities it oversees (Shepherd & You, 2020). One specific regulatory capture is the “Revolving Door,” where politicians or public employees give preferential treatment or particular regard to their past or future employers to move to different employment (Grafton & Williams, 2020). In such cases, employee grievances and whistleblowing will yield no favorable results because managers, companies, and their regulators are motivated by their interests and may sometimes affect safety and quality standards. This may compromise public safety and welfare and is an ethical concern.

While there has been a tremendous amount of research on the downfall of companies due to fraud, corporate mismanagement, and ethical wrongdoings, corporate rigidity in the redressal of employee complaints is relatively unexplored. This paper studies employee grievance redressal as a vector for the success of an organization and how it can lead to a safer environment for the public, stakeholders, and the company itself. It also uses a case-based approach to examine the role of a company’s influence on regulatory bodies in the ignorance of safety-related grievances raised by employees. The case chosen for this study, which perfectly encapsulates the problems of employee grievance redressal mechanisms, regulatory capture, and revolving doors, is the case of Boeing Commercial Airplanes (henceforth Boeing). The case of Boeing is a typical example of a profit-motivated enterprise that suffered massively due to the ignorance of employee grievances, regulatory capture with the Federal Aviation Administration (FAA), and a revolving door of employees between Boeing and the FAA.

## Case Background

The late 2018 and early 2019 crashes of two 737-MAX passenger planes and the subsequent grounding of all 737-MAX aircraft resulted in the questioning of Boeing’s corporate culture (Herkert et al., 2020). The explosions involving the Challenger Space Shuttle in 1986 and Columbia Space Shuttle in 2003, the Deepwater Horizon Oil Spill of 2010, the Ford Pinto case of the 1970s, and the ignition switch scandal involving General Motors in 2014 are engineering disasters that are similar to the case of the Boeing 737-MAX; they all appear to have been caused, in part, by organizational flaws involving poor communication and failure to address safety concerns expressed by employees, which ultimately leads one to question the engineering ethics of these industries. Organizational flaws involving communication gaps, regulatory capture, and a revolving door between the industry and its regulators are common across industries, and a case-based approach could lead one to arrive at some generalized policy recommendations and conclusions.

### The Accidents

The Boeing 737-MAX was born out of the competition against the Airbus A320-NEO, a “lean and clean” short-haul aircraft (Herkert et al., 2020). Although there are other manufacturers, Airbus and Boeing enjoy a duopoly in the commercial aviation manufacturing market (Ibsen, 2009). The fourth generation of the 737 line, the 737-MAX, was introduced by Boeing in August 2011. The aircraft received initial deliveries in May 2017 and soon entered commercial service. After departing Jakarta on October 29, 2018, a Lion Air 737-MAX plane crashed into the Java Sea, killing all 189 on board (Abeyratne, 2020). According to initial inquiries, the aircraft crashed due to a broken flight-control mechanism and erroneous cockpit data. In response to the crashes, the 737-MAX pilots’ operating manuals were updated with instructions on dealing with faulty cockpit data (Ahmed et al., 2019; Jong & Broekman, 2021). On March 10, 2019, a few months after the first crash, an Ethiopian Airlines 737-MAX airliner carrying 157 people crashed six minutes after taking off from Addis Ababa, killing everyone on board (Herkert et al., 2020).

### The Investigation

The “Manoeuvring Characteristics Augmentation System,” more commonly known as MCAS, is the culprit of both crashes (Qin & Wittmann, 2019). However, early investigations into the mishaps could not draw definitive conclusions regarding Boeing’s systems or aircraft (Naor et al., 2020). The MCAS is automatically engaged when the autopilot is disengaged or when the Angle of Attack (AoA) is high (Demirci, 2022). Suppose the angle of attack measurement deviates from the desired limit. In that case, the MCAS will automatically activate itself unless the pilot engages the CUTOUT switch, which turns off the stabilizer trim’s automatic control, or the pilot overrides the system with a manual trim setting (Johnston & Harris, 2019). The MCAS is claimed to have instructed the aircraft’s flight systems to continually descend in response to bogus data from the AoA sensors (Porter, 2020). Boeing did not reveal anything about the MCAS in the 737-MAX pilot guide manual or its supplemental instruction following the first crash (Englehardt et al., 2021). Aviation regulators worldwide ordered the immediate grounding of all Boeing 737-MAX planes. On April 4, 2019, Boeing announced that the 737-MAX crashes involving Lion Air and Ethiopian Airlines involved the MCAS (Boeing, 2019). According to MacArthur (2020), in 2016, one of Boeing’s test pilots for the 737 program warned a coworker about MCAS, citing various issues. The same problems were discovered during the post-crash investigations of the two 737-MAX aircraft. The test pilot made it clear that MCAS was unintentionally activating. This was in contrast to claims made by Boeing officials that it was not intended to engage within the aircraft’s “normal flight envelope,” which justified its exclusion from the 737-MAX’s Standard Operating Procedures (SOPs) (He et al., 2020). Furthermore, according to Appicharla (2023), official reports did not mention MCAS operations and design with much elaboration.

### Key Players

While the FAA is in charge of establishing laws and executing them (such as the federal aviation regulations), the National Transportation Safety Board (NTSB) serves as a neutral arbitrator to give pilots and other airmen access to an ostensibly objective assessment of the FAA’s enforcement decisions (Arendt, 2022) Historically, the NTSB has suggested various changes to the FAA regarding various technical aspects of aircraft overseen by the FAA. However, a few of the major proposals have not been adopted; the relationship between the two entities can be seen as somewhat adversarial (Cobb & Primo, 2003). The corporate relationship between the NTSB and Boeing is slightly unclear in the present literature; however, regarding the revolving door, the NTSB has an unequivocal stance. According to Fielding et al. (2012), when board members and personnel transition to or from the private sector, the NTSB pays particular attention to potential conflicts of interest. For instance, a newly hired NTSB employee from Boeing will not be allowed to serve as the lead investigator on an accident involving a Boeing aircraft during the first year of their employment (a “cooling off” period). They may never be permitted to participate in NTSB investigations of aircraft components they were solely in charge of while working at Boeing. However, it is crucial to note that the NTSB has no regulatory authority and is only responsible for conducting investigations for transportation-related accidents (including commercial aviation accidents) and making safety recommendations (Baxter, 1995; Fielding et al., 2012).

### Grievance Redressal and Whistleblowing at Boeing and the FAA

Whistleblower policies (WBPs) describe the company’s strategy for handling unethical and illegal workplace behavior (Bramstedt, 2022). According to Hassink et al. (2007), many businesses have implemented whistleblowing policies since the Sarbanes-Oxley Act was first introduced in the United States in 2002. In the United States, regulatory agencies rely on whistleblowers to detect corporate misconduct (Heese et al., 2021). However, none of these measures or regulatory agencies will effectively encourage corporate whistleblowing if whistleblowers do not have practical and meaningful access to them (Lombard, 2020). Establishing an effective corporate governance system is essential to avoid external whistleblowing, which may damage the company’s reputation (Andon et al., 2018; Smaili & Arroyo, 2022). This, in turn, makes it crucial for companies to streamline their communication channels and attempt to give significance to employee voice. In this regard, the comments of Lewis (2011) on grievances and whistleblowing are noteworthy: “Workers who are not satisfied with how their grievance has been handled may be protected if they take it to an external body. In order to avoid such an eventuality, employers will need to identify clearly what matters should be processed as a grievance and how that relates to any whistleblowing procedure.” In this regard, this study treats “grievance” as any issue—personal and work-related—arising in the place of work, which is to be redressed by the company. In this study, whistleblowing is defined as exposing these grievances to an internal or external body or agency.

Various complaints were registered by multiple employees, assembly line workers, engineers, quality testers, and various internal whistleblowers against the viability of the Boeing 737-MAX (Pasman, 2021). This leads one to question the effectiveness of the grievance redressal system at Boeing. To gain some insight into this, one has to go back to 2009, when the FAA implemented the Organization Designation Authorization (ODA) program in Boeing to standardize its oversight of Boeing and delegate functions on its behalf (Willis, 2020). The ODA permits a company to carry out specific tasks for the FAA on engineering, production, operations, airworthiness, and maintenance (Federal Aviation Administration, 2018). According to the U.S Department of Transportation (2021), the FAA’s Boeing Aviation Safety Oversight Office comprises 47 FAA employees and oversees the duties delegated to Boeing under the ODA program. There are roughly 1,500 Boeing-designated ODA unit members in the Boeing ODA unit. This means that for every 30 Boeing employees in the ODA, there was only one FAA employee. According to Winkeler et al. (2022), the findings of Boeing’s internal survey from 2016 identified several reasons why there was undue pressure, including schedule pressure, which is brought to light by cost considerations, lack of knowledge on the part of Boeing management regarding their ODA roles, a lack of communication between Unit members and ODA management, and the separation of company and ODA roles. This puts pressure on the members of the Boeing ODA Unit to accept items or confirm compliance with regulations without giving themselves enough time to conduct a review, going against their judgment and experience and possibly jeopardizing the safety of the airplane.

It is interesting to note how employees at Boeing communicated. Following are excerpts from *Flying Blind: The 737-MAX Tragedy and the Fall of Boeing* by Robison (2021), which give some insights into the communication mechanism in the MAX project:

The MAX involved thousands of people spread across offices at Boeing and suppliers around the world, making tens of thousands of individual decisions. Boeing had computer software called DOORS that was supposed to keep everyone around the world updated instantly on the status of each change. It was like an Excel spreadsheet on steroids, with cells that turned red and also sent an update to the finance staff when critical project milestones were breached. (Robison, 2021, p. 130)

In practice, people tended to communicate the way they do in offices everywhere—with emails, instant messages, and phone calls. Whoever yelled loudest got what they wanted, said one person who worked directly with the engineers who coded the MAX software. When it came to fulfilling the FAA’s requirements, the regulator was just one more constituency to satisfy. And not a particularly forceful one. In dealing with the agency’s specialists, Boeing’s engineers came up with what was called ‘the drawer full of paper’ technique. ‘If you can just inundate them with information it makes them go away’. (Robison, 2021, p. 131)

The program’s chief engineer, Michael Teal, reported to Leverkuhn (general manager the MAX project), but none of the fifteen hundred engineers who worked on the plane reported directly to him. They answered to business-unit leaders. (Robison, 2021, p. 131)

In light of the above statements, it is evident that there was some kind of formal communication system at Boeing in the form of the computer software called “DOORS.” However, in practice, the communication system at Boeing appears to have been quite chaotic. Instead of working together towards a common goal, they felt that the FAA was a burden, and employees overloaded them with information to eliminate them. Furthermore, there seems to have been a rigid chain of command; none of the 1,500 engineers who worked on the 737-MAX reported to the general manager of the MAX project. There was a lack of flexibility in the lines of communication up the chain of command, which eventually resulted in regulatory non-compliance.

### Regulatory Capture and the Revolving Door Phenomenon

Dal Bo (2006) gives two definitions of regulatory capture. The narrow definition states that regulated monopolies exercise control over their regulators. The broad interpretation states that certain entities influence the behavior of regulatory agencies in diverse areas, such as legislation, finances, and taxation. Academics view regulatory captures as an institutional pathology because regulators permit regulated organizations to pursue their self-interest or even leverage regulation to their ends at the expense of the public (Downer, 2010). A significant cause of regulatory capture is the cozy relationship between private entities and their regulators (Makkai & Braithwaite, 1992). The phrase “revolving door” refers to the practice in which leaders of governmental agencies enter the industry they have governed after serving their appointed tenure in office (Brezis, 2017). In the case of Boeing and the FAA, Hoppe (2011) states that the FAA is prone to capture and that the interests of a regulated entity can be elevated above the FAA’s priority of safety. The FAA has been found to favor the industry’s interests over passengers’ safety in the past (Nader & Smith, 1994). Following is an excerpt from Rubinstein Reiss (2012, pp. 573–574) from a case involving Southwest Airlines:

In 2007, a large number of Southwest Airlines planes were found to be in violation of an airworthiness directive, and in some cases, parts were showing fatigue cracks (such cracks themselves are not an immediate problem, but their presence is the first sign of the approach of catastrophic failure). Apparently, an FAA inspector warned of the cracks as early as 2003, but nothing was done because of the close relationship between Principal Maintenance Inspector Douglas Gawadzinski and senior management at Southwest Airlines. The connections were so close that FAA personnel allowed Southwest to fly problematic aircraft and even warned Southwest in advance of upcoming inspections.

The excerpt suggests that the relationship between the FAA and the industry is quite concerning. Jakubiak (1997) indicates that the protection of airline safety on the one hand, and the “fostering” of successful air commerce, and consequently, the promotion of airline profitability, on the other, were two interests that might occasionally, if not always, be in conflict, were given to the FAA from the beginning. Boeing’s role in contributing to the revolving door between itself and the FAA is also significant. Gregg (2019) reports that Nikki Haley, a former member of the Trump administration’s cabinet, had been nominated to join The Boeing Company board while Patrick Shanahan, a former executive, served as interim defense secretary. However, shortly after in 2020, Haley resigned from the Boeing Board due to a disagreement with the CEO of Boeing regarding financial stimulus from the federal government (MacMillan, 2022). Following is an excerpt from Schwellenbach and Stodder (2019) that serves as another example of the existence of a revolving door:

Daniel Elwell, who was appointed to the number two spot at the FAA in June 2017 and became the agency’s acting head in January 2018, has gone through the revolving door himself. After serving as an assistant administrator of the FAA from 2006 to 2008, he left government to become vice president of the Aerospace Industries Association, a top industry lobby funded in part by Boeing, and in 2013, became a senior vice president at an airline lobbying group, Airlines for America, before founding a consulting firm in 2015. Elwell maintained close ties with his former lobbyist colleagues after returning to the FAA, corresponding about policy matters.

Daniel Elwell’s professional path is an example of the revolving door problem that raises systemic questions about regulatory agencies, particularly in sectors as crucial as aviation. It raises concerns about potential conflicts of interest and the objectivity of regulatory decision-making when people move easily between high-level positions in industry associations, corporations, and government regulatory functions. Elwell’s decision to leave the FAA for roles in industry lobbying and later return to the FAA raises the possibility of a close relationship between regulatory organizations and the sectors they regulate. Such factors may weaken the objectivity and rigor of regulatory oversight, endangering public safety. To ensure that choices prioritize safety, it is becoming increasingly clear that regulatory organizations must be transparent, hold themselves to high ethical standards, and protect against industry influence.

## Methodology

This paper employs a case-based approach to finding answers to the research questions. Siggelkow (2007) believes cases can help sharpen existing theories by highlighting and filling research gaps. The current literature provides a theoretical base for various intertwined aspects of corporate culture that may lead to the downfall of a business. The case of Boeing serves as a brilliant lesson on careless regulation, broken employee redressal mechanisms, and poor communication (Clarke, 2020; Englehardt et al., 2021; Johnston & Harris, 2019; Jong & Broekman, 2021; MacArthur, 2020; Pontefract, 2019). The gap identified for this research is that corporate employee grievance mechanisms are rarely observed when studying corporate failure. The qualitative analysis will help investigate the importance of employee grievance redressal and how a lack of redressal of safety concerns can lead to public harm.

This paper involves a qualitative study using secondary data from statements of various stakeholders involved in the Boeing case. The qualitative data will be assessed by the rigor of the arguments presented in the statements before, during, and after the accidents. The sample contains three Boeing employees: one senior manager, one senior engineer, and one aerospace engineer. Furthermore, the sample includes a partner at the law firm Kreindler and Kreindler, which represented the victims of the two accidents. Statements of a congressman and a journalist in the New York Times have also been included. The statements of these experts and whistleblowers have been taken from credible sources and are appropriately cited. The names and designations of the sample are in Table 1. The sample has been selected on a purposive basis to achieve the goal of this study. A purposive sample is used to get maximum information from a small sample size. The study attempts to capture a wide range of perspectives by considering various stakeholders involved in the accidents. The themes for analysis have been selected on a deductive basis based on the research questions this paper focuses on.

**Table 1:** Name and designation of sample

Name	Designation
Ed Pierson	Senior Manager at Boeing
Curtis Ewbank	Boeing Aerospace Engineer
Mike Dostert	FAA Aerospace Engineer
Martin Bickeboeller	Senior Engineer
Peter A. DeFazio	Congressman
Justin Green	Partner at Kreindler & Kreindler (Representative of the Victims)
Natalie Kitroeff	Journalist, The New York Times

*Source: Author*

There are three main themes that we look for in the statements of the sample. The first theme is how Boeing did not take the feedback given by its employees seriously. Englehardt et al. (2021) and Herkert et al. (2020) observe very little communication among the various verticals within Boeing. This study takes these observations a step further and hypothesize that this communication was not taken seriously enough, which is proved by the frequent warning signs, but the crashes still occurring. Secondly, we look at how Boeing’s motive of profit and delivery speed led to them ignoring critical feedback concerning the safety of the aircraft. Englehardt et al. (2021) state that due to the increased demand for the 737-MAX caused Boeing to be preoccupied with efficiency and profitability. We attempt to connect Boeing’s objective of efficiency and profitability with disregard for safety concerns raised by Boeing employees. The third theme analyzed is the “revolving door” and the delegation policy between Boeing and the FAA. Pons-Hernández (2022) defines the revolving-door phenomenon as “the movement of individuals from public offices to private companies and vice-versa.” In the case of Boeing, we observe a revolving door recruitment policy in both Boeing and the FAA, i.e., former FAA employees working at Boeing and former Boeing employees working at the FAA. This revolving door, coupled with a high level of delegation regarding safety standards, leads to blurring over which entity is being regulated and which is the regulator. Furthermore, such a relationship between Boeing and the FAA would also result in ignorance of safety-related grievances made directly to the FAA and would make certification of the aircraft much easier (Herkert et al., 2020).

## Case Discussion

The catastrophes involving the Boeing 737-MAX highlight the failures of the aviation regulatory agencies (Sgobba, 2019). This section will analyze the level of ignorance demonstrated by Chief Executive Officer Dennis Muilenburg and other senior Boeing officials towards the internal complaints about the Boeing 737-MAX. The accounts of seven people from different fields will be taken to conduct a qualitative analysis of Boeing’s failure to address employee grievances, their prioritization of profit over safety, and the revolving door between Boeing and the FAA. The personal statements of these seven persons have been obtained from various reputed sources, such as the minutes of official court proceedings, documentaries, and newspaper reports.

### Lack of Efficient Complaint Redressal

From an engineering standpoint, the MCAS was the culprit of the two crashes. However, upon delving deeper, we find various instances of corporate misconduct and, according to Englehardt et al. (2021), a “toxic organizational climate.” Ed Pierson, a former senior manager at Boeing’s Renton factory, exposed Boeing’s toxic organizational climate. When the working conditions at the Boeing factory deteriorated, Pierson attempted to involve top management, but his efforts were unsuccessful. He also contacted the FAA regarding poor work environment, questionable safety, design and engineering flaws, and ignorant management (Committee on Transportation and Infrastructure, 2019).

I formally warned Boeing leadership in writing on multiple occasions, specifically once before the Lion Air crash and again before the Ethiopian Airlines crash, about the potential risks. Those warnings were ignored. I wrote an email to the 737 general manager advising him to shut down the production line. When I mentioned to him that I’ve seen operations in the military shut down for lesser safety concerns, I will never forget his response, which was, ‘Military isn’t a profit-making organization’. (Pierson, as cited in Committee on Transportation and Infrastructure, 2022, p.72)

The fact that warnings were disregarded and that a request to stop the manufacturing line made because of legitimate safety concerns was ignored shows that Boeing did not take safety concerns seriously or respond to them. The manager’s statement implies that their profit motive came before safety. This emphasis on money over safety is a serious red flag that can result in unethical behavior and tragic accidents, in the case of Boeing. Essentially, there was a feedback mechanism, and there were people at Boeing willing to give feedback to the higher-ups, but they were seldom taken seriously.

Curtis Ewbank, Boeing’s flight control engineer, believed that the 737-MAX MCAS had a severe design flaw because it relied solely on one AoA input (Newsham, 2021). He was adamant that senior management at Boeing was fully aware of the data reliability of the AoA sensor and measures to fix it (U.S. Senate Committee on Commerce Science and Transportation, 2021). He frequently urged his superiors to put in place a backup mechanism as he expressed worries about the security of the MCAS software on the new 737-MAX aircraft. His bosses, however, ignored his pleadings due to concerns about the financial consequences.

When CEO Dennis Muilenburg states that there was no ‘technical slip or gap’ in Boeing’s design of the 737-MAX, where a single AoA sensor drove MCAS, he makes a false statement; Boeing, the FAA, and a broad industry team were aware of the necessity of detecting invalid source data and preventing its use by downstream systems. The failure to do that in MCAS is unconscionable, and presenting this situation as anything other than a failure is unethical. (Ewbank, as cited in U.S. Senate Committee on Commerce Science and Transportation, 2021, p. 23)

The statements of Pierson and Ewbank support the hypothesis that even though there was communication, senior management did not take feedback seriously enough. Elangovan and Rajendran (2020) state that there is a dire need for an efficient mechanism for listening to employees’ grievances. This is especially applicable to companies dealing in goods that potentially risk the lives of consumers and the common public. According to Zerfass and Volk (2018), companies must have well-established communication chains across departments and between superiors and subordinates.

To monitor and guarantee consistency of the FAA’s supervision program for businesses that issue certifications and undertake specific inspections on behalf of the agency, the FAA created the Organisation Designation Authorization (ODA) Office (Federal Aviation Administration, 2024). In this regard, the statements of Mike Dostert, an FAA aerospace engineer, give us useful insights:

The current [ODA] system is based upon the assumption that the organization within the company can effectively operate as an independent branch within the company that will force the company to comply with regulations. The ODA selects Authorized Representatives (A.R.s), determines proficiency/competency, regardless of turnover in the organization and organizational pressures within the company to meet certification schedules. In addition, the Boeing ODA has a group review of specific issues that has resulted in many A.R.s not wanting to speak up due to a “group think” phenomenon. The fundamental assumptions that form the basis of the ODA are flawed…….The current system puts barriers to open communication with the FAA. In fact, Boeing has an internal requirement that A.R.s must obtain permission to contact the FAA…….Boeing has demonstrated their ODA is not forcing the company to produce a compliant design. (Dostert, as cited in U.S. Senate Committee on Commerce Science and Transportation, 2021, p. 43)

The ODA system is intended to operate independently within the business and ensure adherence to rules. However, Dostert’s statement implies that this independence was in jeopardy. Organizational demands and the need to satisfy certification deadlines may prevent the ODA from operating autonomously. This lack of impartiality might make communication difficult. The message emphasizes that authorized representatives must first acquire permission to contact the FAA, indicating a barrier to external communication. The transfer of vital information between Boeing and regulatory bodies, which is necessary for guaranteeing safety and compliance, may be slowed down or prevented by this limitation. Overall, Boeing’s implementation of the ODA system is inefficient in enforcing compliance and promoting open communication. This failure implies that the framework and procedures at Boeing did not adequately facilitate the transmission of crucial knowledge and issues.

### Disregard to Safety and Prioritization of Profits

One reason for Boeing’s apparent unwillingness to listen to warnings may be related to the company’s technical and safety culture appearing to change with Boeing’s merger with McDonnell-Douglas in 1997 (Herkert et al., 2020). Boeing’s corporate headquarters moved to Chicago to ensure the compartmentalization of corporate and manufacturing functions. The top management of Boeing was replaced entirely (Useem, 2019). One may argue that there is nothing wrong with wanting to be profitable. However, Boeing lost sight of its responsibility to ensure the safety of their stakeholders and the general public (Englehardt et al., 2021).

Pontefract (2019) states that not only did the company and, arguably, the Federal Aviation Administration (FAA) disregard integrity and safety, but Boeing executives were also motivated by speed to market and speed of re-training their pilots. Martin Bickeboeller, a senior engineer at Boeing, asserts that Boeing has refused to acknowledge violations of federal safety regulations for more than three years following the crashes of two MAX-model 737 airplanes. Concerning the 737 project, Bickeboeller stressed that neither Boeing nor the FAA had adequately responded to his warnings of supply chain non-compliance. Concerns regarding the safety of the 737-MAX that senior engineers raised at the FAA were disregarded when it was being certified (U.S. Senate Committee on Commerce Science and Transportation, 2021).

During 2010–2011, it became clear that in many instances, Boeing management was more interested in a quick resolution without root cause corrective actions, leading to repeated violations of configuration management processes with impact on the product. The culture of regarding procedural violations as an issue to be dealt with when it is convenient is endemic at Boeing and within its executive management…….If Boeing does not acknowledge their failures, how can they correct and improve their corporate compliance and internal culture?! Boeing’s culture of dealing with issues Boeing perceives [to be] “only a violation of regulations” but “probably not a direct product safety issue” is a dangerous culture not conducive to the proper safety of aerospace products. (Bickeboeller, as cited in U.S. Senate Committee on Commerce Science and Transportation, 2021, p. 16)

There is a systemic problem at Boeing when it comes to dealing with safety (Appicharla, 2023). The top management was only interested in results—they wanted a speedy process and swift corrective action. Their disregard for safety was present before and after the Lion Air crash (Herkert et al., 2020; Johnston & Harris, 2019). In the five months between the Lion Air crash and the Ethiopian Airlines crash, many more instances of disregard for the flying public’s safety were discovered. To make matters worse, Boeing appears to have deceived the FAA Aircraft Evaluation Division (AED) about the MCAS and how it impacted the flight control system of the 737-MAX (Department of Justice, 2021). Due to their dishonesty, a crucial document released by the FAA AED was missing information concerning MCAS, which in turn prevented its inclusion in airplane manuals and pilot training materials. This reveals a rather casual attitude towards the safety of the aircraft and the flying public, which did not change even after the first crash. Pierson highlights the relaxed approach of Boeing’s senior management after the first crash in the following extract:

There were at least 13 other reported safety incidents involving new 737-MAX airplanes……that were all manufactured at the 737 factory in Renton, Washington during the same period of time. Most shocking of all, 11 of these 13 safety incidents occurred in the five months between the Lion Air crash and the Ethiopian Airlines crash. That is two safety incidents per month. So, at a time when Boeing and the FAA should have been operating at an extremely heightened sense of awareness after the Lion Air crash, the MAX continued to average two safety incidents per month for the five months leading up to the Ethiopian Airlines crash. At this rate, if the MAX had not been grounded in March 2019, there could have been another 42 safety incidents involving airplane systems (other than MCAS) by December 2020—which means a correspondingly higher probability of another fatal accident. (Pierson, as cited in U.S. Senate Committee on Commerce Science and Transportation, 2021, p. 19)

The cited series of safety mishaps involving Boeing 737-MAX aircraft is extremely unnerving and speaks volumes about a flagrant disrespect for safety issues and a concerning preference for profits over the flying public’s well-being. These events, which persisted at an alarming rate even after the Lion Air crash, cast doubt on Boeing’s dedication to resolving the underlying causes of safety problems. It implies a worrisome lack of urgency and thoroughness in addressing crucial safety problems when it should have been of the utmost importance to take prompt corrective action. This problem, however, was not limited just to Boeing. The FAA is equally accountable for allowing the 737-MAX to fly even after the first crash, even after so many problems were discovered. The FAA’s commitment to safety comes under the spotlight using the statements of Congressman Peter DeFazio. DeFazio was chosen in 2019 to lead the Committee on Transportation & Infrastructure’s investigation of the Boeing crashes. The committee oversees various agencies involving national defense and emergency management.

I was very frustrated by the FAA. They were way less cooperative than Boeing. They refer to the regulated entities as customers. No, they’re not your customers. They are a private interest that will sometimes cut corners to get a competitive edge, and sometimes those corners kill people. During this investigation, we found out something that I think changed everything. After the first crash was a report that the FAA produced about the MCAS system which said that unless the MCAS was modified, 15 of these planes will crash during the life of the fleet. We’ve never allowed anything like that into the air before in history. You don’t do that. Who saw that report? How was it circulated? Why didn’t they then act, to ground the planes, as opposed to waiting until another plane full of people crashed? (DeFazio, in Amer and Mullick, 2022, 1:07:00)

The gravity of the matter has increased with the disclosure of a report from the FAA on the MCAS system. This analysis is alarming because it states that 15 aircraft equipped with the MCAS technology may crash throughout the fleet’s lifespan if no changes are made. It highlights the necessity of prompt regulatory action when solid evidence of possible harm exists. Waiting for further accidents before taking action to protect public safety is a risky strategy that goes against the regulating body’s primary responsibility. Even though the FAA knew the 737-MAX was not airworthy after the first crash, they did not ground the aircraft (Demirci, 2022). The availability of the report, who had access to it, and why prompt action wasn’t taken are crucial questions.

### Revolving Door between Boeing and the FAA

Ralph Nader popularised the notion that regulatory agencies become captives of industry in the 1970s. He argued that this happened because former business executives held influential positions in government organizations tasked with regulating business, but perhaps more importantly, that regulators are lured by the possibility of moving to more lucrative positions in the industries they were tasked with overseeing (Makkai & Braithwaite, 1992). This is called the revolving door phenomenon. It has been hypothesized that regulatory choices may be biased because many regulators originate from or finish up in the industry (Dal Bo, 2006).

Justin Green is a partner at Kreindler & Kreindler, the law firm representing the victims of the Ethiopian crash. He is the co-chair of the “Plaintiff’s Executive Committee” for the case. He has been featured in numerous news segments regarding the two 737-MAX crashes. He is considered an aviation law expert and an important aviation analyst for the Cable News Network (CNN). Green explains the thin wall that exists between Boeing and the FAA in the following excerpt:

The Wall between the FAA and the industry is a very low wall. There’s a lot of back-and-forth movement. It really often is a revolving door. People work for Boeing and then the FAA, and you also see people at the FAA looking to go into Boeing. And what ends up happening is the FAA becomes part of the Boeing team. (Green, in Amer and Mullick, 2022, 1:06:30)

FAA leaders have come from the aviation industry and aviation lobbying groups so often there is now a de facto revolving door between the two. Former FAA administrators with deep industry ties have used their roles as regulators to push industry interests. The FAA has sided with aviation defendants in civil cases maintaining that courts must not hold the defendants liable for failing to comply with state products liability laws, but rather that aviation manufacturers need only comply with federal minimum standards. Instead of promoting the highest level of safety, the FAA could potentially reduce safety and cause aviation victims to lose their rights to seek remedies after the crash of defective aircraft. (Green, 2023)

The passages draw attention to a troubling revolving door situation involving the FAA and the aviation sector. The frequent personnel transfers between the regulatory body and industry may jeopardize the FAA’s regulatory independence. Key FAA jobs may be prioritized by those with backgrounds in the business, potentially at the expense of safety and consumer rights. This calls into doubt the FAA’s capacity to respect victims’ rights and enforce strict safety regulations. The revolving door scenario could jeopardize the agency’s neutrality and efficacy in ensuring aviation public safety because it blurs the borders between the regulator and the regulated. Natalie Kitroeff, a New York Times reporter, was a member of the reporting team that published articles on the Boeing 737-MAX disaster. She explains how, over time, the so-called “revolving door” between Boeing and the FAA resulted in a somewhat “cozy relationship.” Both entities were casual in the certification process of the 737-MAX and were motivated by profits and delivery speed (Herkert et al., 2020).

The airplanes are part of the story, but so are the regulators. The FAA regulated Boeing, in part, with a handful of Boeing employees, whose pay checks came from Boeing, but whose jobs were to represent the interests of the FAA. It’s a decades-old arrangement known as ‘delegation’ that allows federal agencies to give oversight powers to the companies they regulate. In the beginning, there was a really good reason for this. The FAA was certifying things that made no sense; to have them certify every single exit sign or bathroom sign or paint. The issue that many of the FAA employees that we talked to had was that it went way beyond bathroom signs. Over time, Congress passed laws that pushed the FAA to hand over the responsibility for more and more tasks to the company, to Boeing. With this level of delegation between the company and the FAA, it became hard to understand who was working for whom. (Kitroeff, 2021)

The ‘delegation’ relationship between Boeing and the FAA that has been described indicates an unsettling entwining of interests and responsibilities that raises concerns about regulatory capture and the revolving door problem. The delegation system, which was first created to simplify the regulatory procedure for practical reasons, has developed to grant regulated enterprises a wide range of authority. This trend makes it more difficult to distinguish between the genuine allegiances of the regulator and the regulated entity. In such a system, there is a risk of regulatory capture, where the regulator puts the interests of the sector it regulates before public safety. The revolving door problem makes the situation more challenging, which is exemplified by former FAA officials moving into the industry they previously oversaw. It highlights the possibility of conflicts of interest since people who have held regulatory positions in the past might still have strong relationships with their old companies. This might lead to an environment where business interests could significantly sway regulatory decisions, endangering accountability and safety.

The observations made from the statements of Green and Kitroeff indicate that Boeing highly influenced the FAA because of Boeing’s position in the international and domestic markets, which is in line with the observations made by Downer (2010). However, There needs to be a tighter mechanism for the quality assessment of commercial aircraft. The FAA put millions of lives at risk because of their ignorance and lack of attention.

## Recommendations

Transferring important knowledge and expertise between regulators and the industry is possible thanks to the “revolving door” phenomenon. Establishing policies that guarantee some exclusivity and moral behavior in managing business-related issues is necessary, nevertheless. Adopting a system similar to the National Transportation Safety Board (NTSB) that imposes a cooling-off period, or possibly even a lifetime restriction on people for matters related to their former employers, could be one potential solution for the FAA to avoid future accidents. This strategy would support minimizing conflicts of interest, preserve the fairness and objectivity of regulatory judgments, and still permit knowledge sharing.

In response to the flawed ODA mechanism, the FAA appointed an expert panel to review procedures and safety cultures at Boeing. Although the ODA system still exists, the expert panel is a step in the right direction. Furthermore, adding another layer of insulation against potential malpractice, the Associate Administrator for Aviation Safety at the FAA now has direct authority over the ODA Office (Federal Aviation Administration, 2024). Instead of complete delegation, layers of protection and establishment of accountability must be added to safeguard the public against any mishap, as in the case of Boeing. There should always be a “Due Process” that must be followed in case of any regulatory delegation (Volokh, 2014). Communication lines should be made to be more flexible. Although management literature emphasizes a chain of command to enhance worker outcomes, it is, after all, a chain of command and not a chain of feedback. A chain of feedback, or rather, a more flexible feedback mechanism wherein employees feel safe to blow the whistle freely regarding problems they face, should be the new norm.

A thorough set of policy recommendations is necessary for Boeing to improve employee grievance redressal. This means establishing an unbiased, independent office to strengthen whistleblower protection and address employee complaints. In this regard, a significant contribution was made by Fischbach and Gilbert (1995), who suggest that organizations create the role of “Ombudsman.” This person would play a variety of responsibilities, including providing confidential advice on basic, applied, or clinical research issues and acting as a facilitator for persons desiring to follow a formal grievance process. In terms of grievance reporting, the case of Microsoft is an interesting one. The “Microsoft Integrity Portal” encourages anonymous reporting through Email, Phone, Fax, Mail, and an external hotline (Microsoft, 2023). It also establishes “Due Process” steps for employees and managers to raise and address these grievances.

Safety should be put above profits in a fundamental shift in organizational culture, and employees should actively participate in safety choices. Internal safety checks, open reporting, and strict regulatory compliance are essential. While extensive safety training and feedback mechanisms must be put in place, transparency can be improved by external oversight and public reporting. Committing to continuous improvement means that issues are detected and turned into significant safety improvements, strengthening Boeing’s commitment to worker safety and well-being.

## Conclusions

The problems discussed in the paper, as demonstrated by the example of Boeing, go beyond the purview of any one company or sector. They highlight the widespread systemic issues with business environments and international regulatory systems. For organizations and regulatory authorities across many industries to be safe, ethical, and accountable, these issues must be addressed. The analysis emphasizes the urgent need for Boeing to own up to its mistakes, fix its corporate compliance issues, improve its employee grievance redressal mechanism, and promote a culture that puts aerospace product safety first. The tragedies involving the Boeing 737-MAX have brought to light the possible repercussions of a culture where profits come before safety, and the effects might be terrible for the firm and the larger aviation sector. Addressing these cultural concerns and embracing a fresh commitment to security and compliance are essential to Boeing’s future performance and reputation. For this, establishing efficient lines of communication where such communication is considered with utmost seriousness is vital.

The paper contributes to a more comprehensive understanding of Boeing’s weak grievance redressal and regulation and how it ultimately led to a loss of life. The Boeing case exemplifies the significance of robust internal grievance mechanisms that prioritize safety and transparency over profits. Similar problems exist worldwide in various industries but only come to light after tragedy strikes. The problem, however, is omnipresent. This study highlights the need for real-time risk management, clear communication channels, and top-down governance to handle employee concerns properly, thus preventing lapses in safety and ethical standards.

Engineers, engineering instructors, managers, ombudsmen, and human resource practitioners may learn a lot from the Boeing 737-MAX case, highlighting the ethical responsibilities of the job. It emphasizes that safety should never be sacrificed for cost savings or to meet rigid delivery deadlines because such decisions are unethical. As Herkert et al. (2020) state, the 737-MAX decisions made by Boeing, particularly those involving MCAS, are determined to be unethical using a variety of ethical frameworks. This emphasizes the necessity for strong regulatory oversight to protect public safety and the profound junction of engineering ethics, responsible innovation, and business ethics. It also highlights the significance of adaptable communication in encouraging open exchange of concerns.
